# Extracellular Acidosis Differentially Regulates Estrogen Receptor β-Dependent EMT Reprogramming in Female and Male Melanoma Cells

**DOI:** 10.3390/ijms232315374

**Published:** 2022-12-06

**Authors:** Silvia Peppicelli, Jessica Ruzzolini, Matteo Lulli, Alessio Biagioni, Francesca Bianchini, Adele Caldarella, Chiara Nediani, Elena Andreucci, Lido Calorini

**Affiliations:** 1 Department of Experimental and Clinical Biomedical Sciences “Mario Serio”, University of Florence, 50134 Florence, Italy; 2 Tuscany Cancer Registry, Clinical Epidemiology Unit, Institute for Cancer Research, Prevention and Clinical Network (ISPRO)-Florence, 50139 Florence, Italy; 3 Center of Excellence for Research, Transfer and High Education DenoTHE, University of Florence, 50134 Florence, Italy

**Keywords:** extracellular acidity, estrogen receptor β, epithelial-to-mesenchymal transition, NF-κB, human melanoma

## Abstract

Clinical outcomes of melanoma patients pointed out a gender disparity that supports a correlation between sex hormone activity on estrogen receptors (ER) and melanoma development and progression. Here, we found that the epithelial-to-mesenchymal transition (EMT) of melanoma cells induced by extracellular acidosis, which is a crucial hallmark of solid cancers, correlates with the expression of ERβ, the most representative ER on melanoma cells. Extracellular acidosis induces an enhanced expression of ERβ in female cells and EMT markers remain unchanged, while extracellular acidosis did not induce the expression of ERβ in male cells and EMT was strongly promoted. An inverse relationship between ERβ expression and EMT markers in melanoma cells of different sex exposed to extracellular acidosis was revealed by two different technical approaches: florescence-activated cell sorting of high ERβ expressing cell subpopulations and ERβ receptor silencing. Finally, we found that ERβ regulates EMT through NF-κB activation. These results demonstrate that extracellular acidosis drives a differential ERβ regulation in male and female melanoma cells and that this gender disparity might open new perspectives for personalized therapeutic approaches.

## 1. Introduction

In the last decade, melanoma, a highly aggressive and heterogeneous tumor, has exhibited an increased incidence. More than ~50% of melanoma lesions are characterized by point mutations of BRAF (V-raf murine sarcoma viral oncogene homolog B1), and the most common mutation displayed is a valine to glutamic acid substitution (V600E) [1]. Since BRAF regulates the MAPK pathway, this genetic trait became the target for specific therapy. Nevertheless, it is well recognized that the multiple phenotypes characterizing melanoma lesions depend not only on gene expression but also on newly activated transcriptional programs. This means that changes in transcriptional regulators are responsible for a continuous adaptive phenotype defined as tumor cell plasticity. One of the most intriguing factors that are capable of modulating both the gene expression and the transcriptional machinery in melanoma is sex [2]. Indeed, men have a higher risk of developing melanoma compared to women, and often their prognosis is worse [3]. Further, men have a higher probability of developing melanoma lesions during their lifetime compared to women (1.72% vs. 1.22%). Localized melanoma in women showed a lower tendency to metastasize, resulting in a better survival rate when compared to that of men [4]. These differences might be based on behavioral characteristics in men, due to their lifestyle (e.g., delay in diagnosis, exposure to the sun); however, even after adjusting for factors affected by these characteristics, men with melanoma still have poorer outcomes than their female counterparts, and sex remains an independent significant survival predictor. Therefore, researchers suggest that there is a biological trait not yet fully elucidated that accounts for the sex-related survival advantage in melanoma. Growing evidence supports a direct correlation between sex hormone levels and melanoma progression [5]. Estrogens exert their effects through estrogen receptors (ER), ERα and ERβ which affect cancer growth in opposite ways: ERα is associated with a positive effect on cell proliferation, while ERβ, the predominant ER in melanoma, is associated with an anticancer effect [5]. Recent studies showed that obese men with melanoma show better survival and respond better to targeted therapy compared to those who have a normal body mass index (the so-called obesity paradox) [6]. It is known that obese men have higher levels of estrogen in their blood [7], supporting a protective role of sex hormone/ER activity on disease progression.

The epithelial-mesenchymal transitions (EMT), the acquisition of mesenchymal features from epithelial cells, represents a crucial event of tumor progression, conferring to tumor cells stem-cell-like properties, increasing the ability to survive, infiltrate, and disseminate [8]. Indeed, when epithelial tumor cells undergo EMT, they lose cell polarity and cell-cell adhesion, gaining invasive ability. Among the most important transcription factors involved in EMT, there are Snail, Twist, and Zeb ½. Moreover, EMT is characterized by high levels of vimentin, reduction of E-cadherin, and elevation of N-cadherin [9]. In melanoma, the “phenotype switching” is also dependent on the expression of the Microphthalmia-associated transcription factor MITF, the master regulator of melanocyte development [10]: a high level of MITF keeps melanocytes in the differentiated status, intermediate level of MITF supports proliferation, low MITF expression induces cells invasiveness.

The objective of this study is to elucidate ERβ expression and its role in the epithelial-to-mesenchymal transition (EMT) switch of melanoma cells of both sexes exposed to one of the most effective pro-tumoral aspects of a tumor’s microenvironment (TME), low pH. Indeed, in several solid tumors, including melanoma, extracellular pH ranges between 6.7–6.9 [11,12]. It is well known that extracellular acidosis promotes resistance to BRAF inhibitors [13], invasion of surrounding tissues, and development of distant metastasis [14], and drives the acquisition of a stem-like phenotype [15] and EMT [14]. The use of primary and established cultures of melanoma cells of both sexes with comparable malignancy allowed us to demonstrate that ERβ protects female melanoma cells from EMT reprogramming in extracellular acidosis, through NF-κB down-regulation.

## 2. Results

### 2.1. ERβ Expression and EMT Reprogramming in Melanoma Cells Exposed to Extracellular Acidosis

We know that the tumor microenvironment generates signaling pathways critical for reconverting tumor cell adaptive phenotype, and that metabolic conditions, such as hypoxia and acidosis, may drive a more aggressive phenotype in tumor cells. 

Before analyzing the influence of a reduced pH on ERβ expression in human melanoma cells, we verified the ERβ protein expression in several male and female melanoma cells, using MCF7 ERβ-positive mammary cancer cells as a positive control (Figure S1).

**Figure 1:**
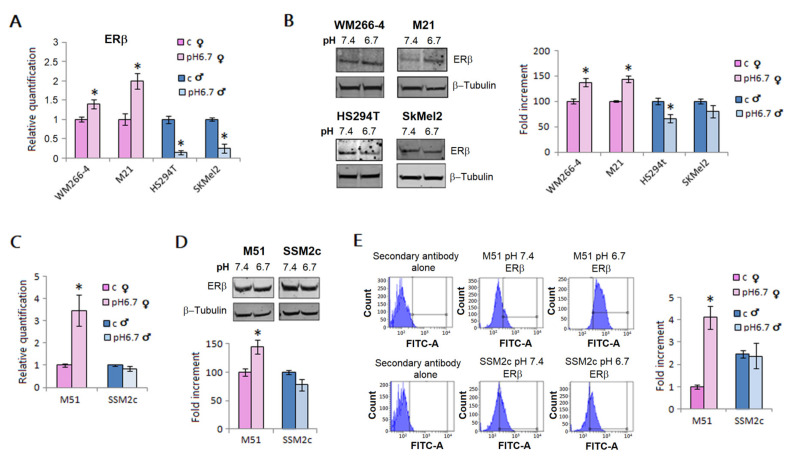
**ERβ expression in melanoma cells of male and female patients.** mRNA (**A**) and protein (**B**) levels of ERβ in female (WM266-4, M21) and male melanoma cells (HS294t, SKMEL2) grown in standard (pH 7.4) or acidic medium (pH 6.7) for 24 h. mRNA level and protein expression of ERβ in M51 (female) and SSM2c (male) melanoma cells grown in an acidic medium for 24 h, performed by Real-time PCR (**C**), Western blotting (**D**), and flow cytometry (**E**). Histograms represent the mean ± SEM of at least three independent experiments. * *p* < 0.05 compared with control cells.

We exposed male and female melanoma cells for 24 h to a pH 6.7 acidified medium. We found that acidity promotes ERβ expression in female WM266-4 and M21 metastatic melanoma cells, whereas, in contrast, its expression is reduced in male HS294T and SKMEL2 metastatic melanoma cells, either at mRNA (Figure 1, panel A) or protein levels (Figure 1, panel B). We also tested melanoma cells isolated from clinically comparable metastatic lesions: female M51 and male SSM2c cells. Using these cells, we were able to strengthen the finding that acidic female cells up-regulate ERβ expression, whereas acidosis was not able to affect ERβ expression in male cells; these results were obtained using real-time PCR (Figure 1, panel C), Western blotting (Figure 1, panel D) and flow cytometry of permeabilized cells (Figure 1, panel E). Moreover, comparable results were found when M51 and SSM2c cells were grown in DMEM without phenol red, one of the most critical components of media with estrogen-like activity (see Supplementary Figure S2).

**Figure 2:**
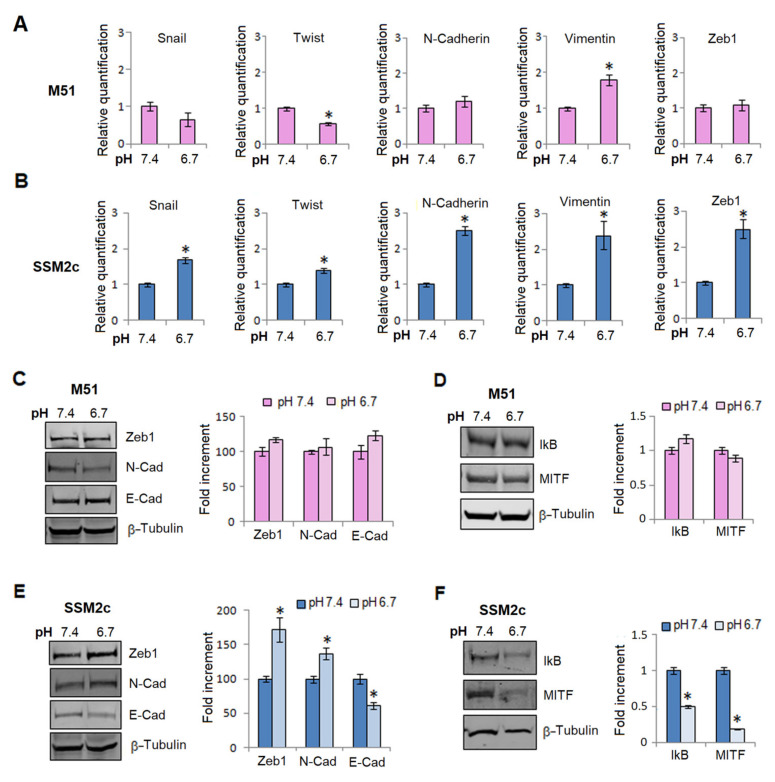
**EMT reprogramming in acidic male and female melanoma cells.** mRNA level of EMT markers of M51 female melanoma cells (**A**) and SSM2c male melanoma cells (**B**) grown in an acidic medium for 24 h. Zeb1, E-cadherin, N-cadherin protein expression (**C**), and IKB and MITF protein expression (**D**) performed by Western blotting of female M51 melanoma cells. Zeb1, E-cadherin, N-cadherin protein expression (**E**), and IKB and MITF protein expression (**F**) performed by Western blotting of male SSM2c melanoma cells. Each band of the western blot was quantified by densitometric analysis, and the corresponding histogram was constructed relative to β-tubulin. Representative Western blot panels on the left. Histograms represent the mean ± SEM of at least three independent experiments. * *p* < 0.05 compared with control cells.

The results encouraged us to continue our investigation and the epithelial-to-mesenchymal transition (EMT) program of primary melanoma cells became the target of our study. As reported in Figure 2, acidic female M51 cells, among the several markers and transcription factors of EMT investigated, showed only an increased amount of vimentin (Figure 2, panel A), whereas acidic male SSM2c cells expressed a clear enhancement of mRNA mesenchymal markers, such as N-cadherin and vimentin, and an increasing amount of Twist, SNAIL, and Zeb1, three of the major transcription factors governing EMT in various tumor cells (Figure 2, panel B). These differences in EMT induction in acidic M51 and SSM2c cells were confirmed by Western blotting analysis of the EMT markers Zeb1, N-cadherin, and E-Cadherin (Figure 2, panel C,E). Indeed, only male melanoma cells showed a significant reduction of E-cadherin and an increased expression of N-cadherin and Zeb1 (Figure 2, panel E), while in female melanoma cells none of the EMT markers significantly changed (Figure 2, panel C).

We also evaluated the expression of IkB, the inhibitor of NF-κB, and the expression of the microphthalmia-associated transcription factor (MITF), one of the most important melanoma transcriptional regulators, which drives a switch toward a proliferating phenotype. While acidic female M51 cells show an unchanged expression of IkB and MITF (Figure 2, panel D), acidic male SSM2c cells express a reduced content of these proteins (Figure 2, panel F). A low level of IkB reveals an increased activity of NF-κB transcription factor as we have already found in acidic cells [14], while the parallel reduction of MITF identifies a switch of melanoma cells from a proliferative to an invasive phenotype (see Supplementary Figure S3, panel A).

Furthermore, female WM266-4 and M21 melanoma cells exposed to acidosis do not modify their proliferation, N-cadherin, and vimentin expression, while male HS294t and SKMEL2 cells show a reduction in cell growth and a significant increase of N-cadherin and vimentin (see Supplementary Figure S3, panels B and C). 

We next tested whether ERβ might have a role in EMT reprogramming.

### 2.2. ERβ as a Potent Inhibitor of EMT in Human Melanoma Cells

Our previous findings allowed us to test whether ERβ might have a role in the EMT switch of melanoma cells of both sexes. To determine possible differences in the regulation of EMT by ERβ in melanoma cells in response to acidosis, we decided to use a double approach constituted by ERβ silencing experiments and through the isolation by flow cytometry cell sorting of low and high ERβ expressing subpopulations. 

**Figure 3:**
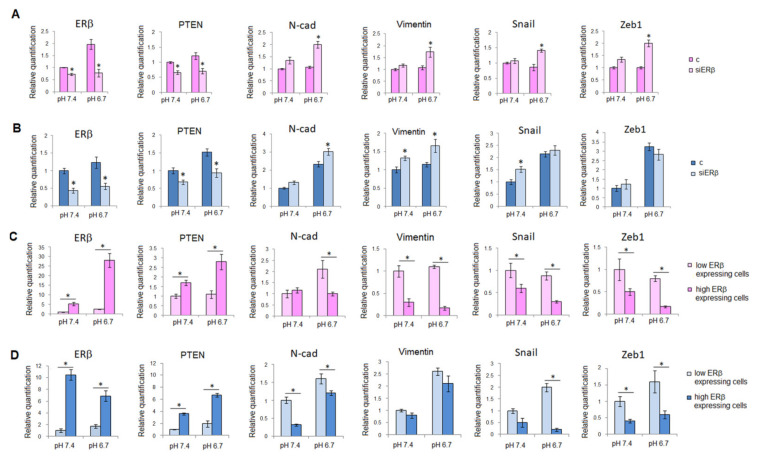
**EMT regulation by ERβ.** mRNA level of ERβ and EMT markers of M51 female (**A**) and SSM2c male (**B**) melanoma cells silenced for ESR2 and grown in standard or acidic medium for 24 h. (**C**) mRNA level of ERβ and EMT markers of ERβ low and high expressing M51 female melanoma cell subpopulations. (**D**) mRNA level of ERβ and EMT markers of ERβ low and high expressing SSM2c male melanoma cell subpopulations. Histograms represent the mean ± SEM of at least three independent experiments. * *p* < 0.05 compared with control cells.

We proved ERβ silencing of melanoma cells by analyzing mRNA and protein expression of ERβ (Figure 3, panels A,B, and Figure S4, panels A,B).

As expected, low pH was able to induce a high expression of ERβ in M51 cells (Figure 3, panel A). After ERβ silencing we observed that in addition to a significant reduction of ERβ also PTEN, a tumor suppressor which can be modulated by ERβ [16], was reduced, while N-cadherin, vimentin, SNAIL, and Zeb-1 increased after acidic treatment, justifying the phenotype drift to a mesenchymal state (Figure 3, panel A). These last findings support the notion that PTEN suppresses EMT in melanoma cells, as reported in mammary tumor cells [17]. When we silenced ERβ in SSM2c male cells, we found a significant reduction of ERβ and PTEN, although the increased expression of EMT-related genes was less evident than in M51 cells (Figure 3, panel B).

In ERβ silenced-non-acidic cells we failed to find any change in EMT markers expression with the only variation of PTEN reduction. This is possible for the already low level of ERβ in female cells. 

As reported in Figure 3 panel C, sorted subpopulations (at low and high ERβ expression; see Figure S4, panels B and C) of female M51 melanoma cells, which were grown either at standard pH or acidic pH, show a corresponding low and high level of ERβ. The high-expressing ERβ subpopulation from acidic cells possesses the most elevated ERβ expression. The same subpopulation at high ERβ expression is characterized by a significant reduction of EMT markers, N-cadherin, vimentin, SNAIL, and Zeb-1 (Figure 3, panel C), associated with an elevation of PTEN. On the other hand, the M51 subpopulations characterized by low expression of ERβ show high expression of EMT markers and low PTEN expression level (Figure 3, panel C). In parallel, we conducted the same experiment (cell sorting) on male SSM2c melanoma cells and we found that the isolated low- and high-ERβ expressing male subpopulations showed a behavior comparable to the female subpopulations (Figure 3, panel D).

**Figure 4:**
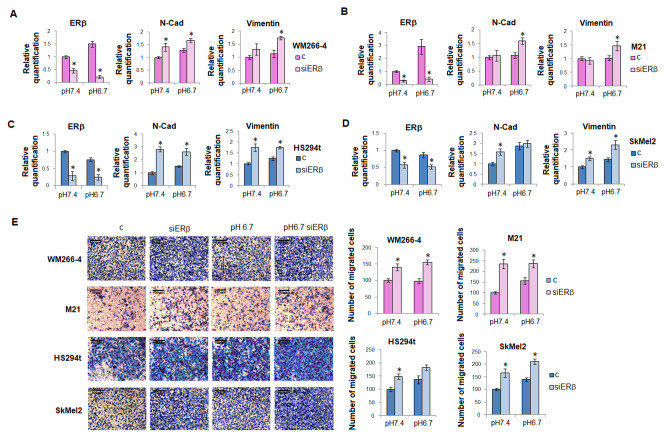
**EMT in ERβ silenced melanoma cell lines.** mRNA level of ERβ, N-cadherin, and vimentin in WM266-4 (**A**), M21 (**B**) female melanoma cells and in HS294t (**C**) and SKMEL2 (**D**) male melanoma cells. (**E**) Invasive ability of male and female melanoma cells silenced for ERβ, grown in standard pH or acidic pH medium. Invasiveness was performed using Matrigel-coated filters and it was measured as a percentage of the control. Data are expressed as means ± SEM of three independent experiments. * *p* < 0.05.

To confirm a causal relationship between ERβ expression and EMT in melanoma of both sexes, we silenced ERβ in female WM266-4 and M21 cells and male HS294t and SKMEL2 cells (Figure S5) and then we exposed these cells to acidosis. After ERβ silencing we observed a rapid increase of N-cadherin and vimentin in both female and male cell lines (Figure 4, panels A,B, and C,D). When we tested cell invasive ability through Matrigel-coated filters we observed that ERβ silenced melanoma cells express a high ability to invade. Moreover, we found that in acidic female melanoma cells, in which the ERβ expression increase correlates with an unaltered level of N-cadherin and vimentin, invasiveness does not change (Figure 4, panel E). Instead, in male melanoma cell lines, where an unchanged level of ERβ in acidosis is associated with an enhanced expression of N-cadherin and vimentin, cell invasiveness increases (Figure 4, panel E). These results strengthen the observation of a tight relationship between ERβ and EMT.

### 2.3. ERβ-NF-κB Crosstalk in EMT of Melanoma Cells 

**Figure 5:**
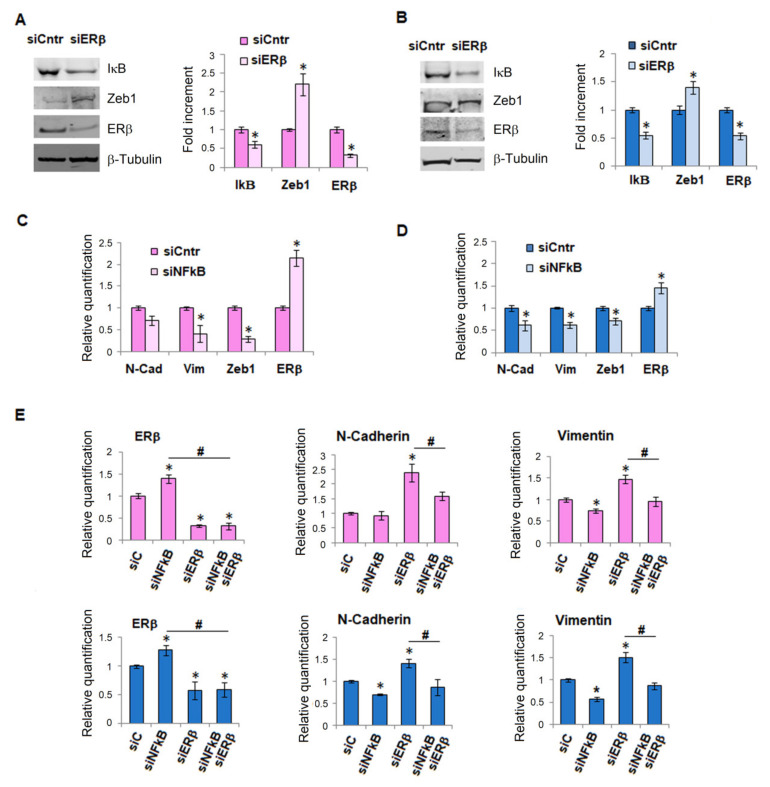
**ER**β**-NFkB crosstalk.** IkB, Zeb1, and ERβ protein expression performed by Western blotting of female M51 melanoma cells (**A**) and male SSM2c melanoma cells (**B**) ERβ silenced. Each band of western blot was quantified by densitometric analysis and the corresponding histogram was constructed relative to β-tubulin. Representative Western blot panels on the left. mRNA expression of ERβ and EMT markers in female M51 melanoma cells (**C**) and male SSM2c melanoma cells (**D**) NF-κB silenced. (**E**) mRNA expression of ERβ, N-cadherin, and Vimentin, in M51 and SSM2c melanoma cells silenced for NF-κB and after 24 h silenced for ERβ. Histograms represent the mean ± SEM of at least three independent experiments. * *p* < 0.05 compared with control cells. # *p* < 0.05.

NF-κB acquires a particular significance in cancer progression and the activation of its signaling constitutes a key event, contributing to tumor development and metastatic dissemination [18]. Since NF-κB also plays a pivotal role in the EMT of cancer epithelial cells, mostly through its stabilization of Snail [19], we tried to understand whether there was a relationship between NF-κB and ERβ. After silencing ERβ both in female and male melanoma cells, we found, in addition to an increase of Zeb1, also a significant reduction of IkB (Figure 5, panels A and B), which leads to NF-κB activation. In parallel experiments, the silencing of NF-κB drives a rapid increase of ERβ and a decrease of EMT markers, N-cadherin, vimentin, Zeb1, both in female and male melanoma cells (Figure 5, panels C and D; see also Figure S6). 

Finally, to establish whether there is a special hierarchy between the two agents, we conducted experiments on ERβ and NF-κB dual silencing. We evaluated by real-time PCR the expression level of N-cadherin and vimentin both in female and male melanoma cells and we found that in double silenced cells the expression of these EMT markers was as low as the level obtained after NF-ΚB silencing. This result indicates that ERβ does not regulate the EMT directly, but rather through its modulation of NF-κB, which remains the main driver of EMT (Figure 5, panel E). This last finding suggests that the anti-oncoprotein ERβ might have some relevance in malignancy. In the case of direct activation of NF-κB, however, the protective effect of ERβ fails, allowing the progression of cancer cells toward malignancy.

## 3. Discussion

To identify possible mechanisms involved in gender disparity frequently observed in cutaneous melanoma, we investigated the role of ERβ in EMT reprogramming of melanoma cells of both sexes upon their exposure to acidic culture media.

It is known that solid tumors, such as melanoma, are frequently characterized by an inadequate vasculature that is responsible for a transient or persistent oxygen deficiency promoting a metabolic adaptation of tumor cells to anaerobic glycolysis under the HIF-1α transcription factor activity. This metabolic switch leads to protons and lactate extrusion, which, in the absence of effective lymphatic support, accumulate and contribute to extracellular acidosis [20]. The extracellular pH of melanoma lesions ranges between 6.4/6.6 to 7.0/7.3 [11,12], and acidosis is associated with a poorer prognosis and aggressiveness in many different types of cancer [21,22,23,24,25,26]. Considering the ability of acidosis to reconvert tumor cells toward an aggressive phenotype, we focused our study on the possible gender disparity of ERβ expression on acidic melanoma cells. ERβ has been reported as the critical ER in melanocytic lesions [27], and De Giorgi et al. described that the level of ERβ was found to be reduced in thicker and invasive melanoma [28,29], supporting the notion that ERβ might have anti-tumor activity in melanoma [5].

We found, quite surprisingly, that while female acidic melanoma cells up-regulate ERβ expression both at mRNA and protein levels, male acidic melanoma cells do not modify ERβ expression (or even reduced it). This behavior was observed in female and male, well-established, melanoma cell lines usually used in gender studies [30] and primary cultures isolated from cutaneous metastatic lesions of a comparable clinical aggressiveness. To verify whether the change in expression of the tumor suppressor ERβ associates with a change in tumor cell phenotype, we studied EMT markers in melanoma of both sexes after exposure to acidosis. It is known that carcinoma cells during their progression toward a malignant phenotype experience EMT, losing epithelial markers and acquiring mesenchymal traits, including the ability to disseminate and stem cell-like features [31]. This is also true for melanoma cells.

The present study indicates that an acidic medium promotes most EMT markers only in male SSM2c cells and not in female M51 cells. An increase in the expression of N-cadherin and vimentin markers and SNAIL, Twist, and Zeb1 transcription factors was detected in acidic male cells, as well as a reduction in E-cadherin, in line with the so-called cadherin shift [32,33]. In acidic female cells, only vimentin expression was higher than that of control cells grown in a non-acidic medium. N-cadherin acquired by male melanoma cells may act as an oncogene and promote local invasiveness [34] and metastatic dissemination [35], while it has been reported that adenoviral re-expression of E-cadherin reduces malignancy [36]. Acidic male cells also showed an evident decrease of IkB, meaning an increase in NF-κB activity. NF-κB may directly up-regulate the transcription of genes involved in EMT and act indirectly through the up-regulation of Snail which, in turn, suppresses the expression of Raf kinase inhibitor protein RKIP (an inhibitor of MAPK and NF-κB pathways) and PTEN (an inhibitor of PI3K/AKT pathway) [37]. Similarly, other evidence highlights that NF-κB also regulates the EMT transcription factors in breast cancer cells [38]. A further indication that acidic male cells move towards an invasive phenotype is the reduction of MITF, whereas the unchanged level of MITF in acidic female cells strengthens the expression of a proliferative phenotype that can potentially be targeted by therapy. 

Now to understand whether ERβ may have a role in the EMT switch in melanoma cells of both sexes, in particular in acidic female melanoma cells expressing an increased level of ERβ in combination with a lack of EMT reprogramming, we used two approaches: (a) ERβ silencing assay and (b) cell sorter flow cytometry isolation of low and high ERβ expressing subpopulations. Both approaches revealed that ERβ has a role in EMT marker and transcription factor regulation. These findings are particularly evident in acidic female melanoma cells, where changes in all markers of EMT were inversed with respect to ERβ expression. Thus, female melanoma cells with high expression of ERβ show a reduction in N-Cadherin, vimentin, Snail, and Zeb1 and characteristically a parallel increase in PTEN expression; while in the ERβ silencing experiments, markers of EMT rise and PTEN decreases. In acidic male melanoma cells, the correlation between ERβ expression and markers of EMT was incomplete, although it is suggestive of an inverse correlation between the level of ERβ and EMT. In agreement with Zhang et al. [39], who demonstrated that silencing ERβ promotes the invasion and migration of osteosarcoma cells, we found that melanoma cells silenced for ERβ increase their invasive ability. 

We point out that, while in the present study we used parental heterogeneous cell lines, in our previous publications, in which female melanoma cells undergo EMT [14,15], we used stably aggressive subpopulations selected through in vitro (three-month acidosis, e.g., chronic acidosis [15]) or in vivo (colonization following intravenous injection of tumor cells [14]) procedures. Indeed, as revealed by cell sorter flow cytometry analyses, the parental female melanoma cells, grown for 24-h in acidic media, maintain their heterogeneity, even after acidosis exposure, disclosing stably unresponsive ERβ cell subpopulations expressing an EMT phenotype.

Finally, our experiments of double silencing strengthen the liaison between ERβ and NF-κB revealing an inverse regulation, although the dominant driver of EMT remains NF-κB. The ability of ERs to repress the transcriptional activity of NF-κB has been well studied [40,41,42], but the ability of NF-κB to regulate ERβ expression has never been described.

Overall, ERβ in addition to representing one of the most important prognostic factors for melanoma [29], is described in the present paper as inversely associated with EMT in acidosis. Due to the limited number of cell lines used, the establishment of an ERβ related new mechanism of sex disparity will require a greater extended epidemiological study of ERβ-dependent EMT in female and male patients. This approach might be able to improve the monitoring and treatment of patients at high risk for disease progression. Seleiro D et al. [43] showed an enhanced presence of mucin-depleted foci, a marker of a progressive lesion in ERβ knockout mice, signifying a protective activity of this estrogen receptor in colon cancer. Furthermore, the observation of Zhao Xiaojian et al. [44] is quite interesting whereby some sort of signaling elicited by hypoxia controlling proliferation and apoptosis in cardiac fibroblasts is stimulated in a gender-specific manner. 

Thus, understanding the mechanisms in which ERβ is involved may open up new perspectives for personalized melanoma approaches, aimed at controlling tumor dissemination and relapse.

## 4. Materials and Methods

### 4.1. Cell Cultures

The patient-derived SSM2c and M51 melanoma cells were kindly provided by Dr. Stecca B., Laboratory of Tumor Cell Biology, Istituto Toscano Tumori (ITT) Italy [30]. In particular, SSM2c cells originate from loco-regional in transit subcutaneous melanoma metastasis of a male patient, and M51 cells are from loco-regional in transit subcutaneous melanoma metastasis of a female patient: the local lesion and patient outcome assured a comparable level of malignancy of the isolated melanoma cells. Experiments were performed also using some of the most commonly used human melanoma cell lines of both sexes: WM266-4 (obtained from American Type Culture Collection (ATCC), Manassas, VA) and M21 (kindly provided by Dr. Antony Montgomery, The Scripps Research Institute, La Jolla, CA, USA), derived from female patients; HS294t and SkMel2 (obtained from ATCC), derived from male patients. The information regarding the cell lines and the patients from which they derive are summarized in Supplementary Table S1. Moreover, using the Devyser Resolution XY kit (Cat. No. 8-A012.2-XY, Devyser, HQ, Hägersten, Sweden), a QF-PCR kit for rapid quantitative analysis of chromosomes X and Y, the Diagnostica Genetica’s team of Azienda Ospedaliero-Universitaria Careggi (University of Florence) verified that HS294t and SKMel-2 cells conserved the Y chromosome, while the SSM2c cells did not show it starting from their early culture passages (for details see Supplementary Figure S7).

The human breast carcinoma MCF7 cell line, which is known to express the estrogen receptor beta, was provided by ATCC and was used as a positive control for ERβ expression.

Cells were cultured in DMEM 4.5 g/l glucose (Cat. no. ECM0728, Euroclone, Milan, Italy), supplemented with 2 mM L-glutamine (Cat. no. ECB3000D, Euroclone) and 10% FBS (Cat. No. ECS0180L; Euroclone). Cells were harvested from subconfluent cultures by incubation with a trypsin-EDTA solution (Cat. No. ECB3052, EuroClone), and propagated every three days. The viability of the cells was determined by Trypan Blue Exclusion test. Cultures were periodically monitored for mycoplasma contamination using Chen’s fluorochrome test [45].

The acidic condition was obtained in vitro by adding HCl 1 N in a complete culture medium to reach pH 6.7 ± 0.1. pH value was monitored by using Orion pH meter 520A-1 at regular intervals during the first hour after the acidification to check the maintenance of the correct pH of the medium. As the pH value was stable, the acidified medium was added to cell cultures and the seal cap was tightly closed to prevent buffering. At the end of exposure, pH was also evaluated to control its maintenance (pH 6.7 ± 0.1). During the long-lasting acidic treatment, no significant death of cells was found [13].

### 4.2. Quantitative Real-Time PCR (qPCR)

Total RNA was prepared using Tri Reagent (Cat. No. T9424, Sigma-Aldrich, Milan, Italy), agarose gel checked for integrity, and reverse transcribed with iScript cDNA Synthesis Kit (Cat. No. 1708891, BioRad, Segrate, MI, Italy) according to the manufacturer’s instructions. Quantitative real-time PCR (qPCR) was performed using the Sso Advanced Universal SYBR Green Supermix (Cat. No. 1725274, BioRad). The qPCR analysis was carried out in triplicate with a CFX96 Real-Time PCR System (BioRad) with the default PCR setting: 40 cycles of 95° for 10 s and 60 °C for 30 s. The fold change was determined by the comparative Ct method using 18S and β-actin as reference genes. Primer sequences are listed in Table 1.

### 4.3. Western Blotting Analysis

Cells were washed with ice-cold PBS containing 1 mM Na4VO3 and lysed in about 100 μL of cell RIPA lysis buffer (Cat. No. 20–188; Merk Millipore, Vimodrone, MI, Italy), containing PMSF (Cat. No. 10837091001, Sigma-Aldrich), sodium orthovanadate (Cat. No. 567540; Sigma-Aldrich) and protease inhibitor mini tablets (Cat. No. A32955, Life Technologies, Monza, Italy) as previously described [15]. Aliquots of supernatants containing equal amounts of protein (50–100 μg) in Bolt LDS Sample Buffer (Cat. No. B0007, Life Technologies) were separated on Bolt^®^ Bis-Tris Plus gels 4–12% precast polyacrylamide gels (Cat. No. NW04122BOX; Life Technologies). Fractionated proteins were transferred from the gel to a PVDF nitrocellulose membrane using an electroblotting apparatus (BioRad). Blots were blocked for 5 min, at room temperature, with Every Blot Blocking Buffer (Cat. No. 12010020, Bio-Rad) and the membrane was probed at 4 °C overnight with primary antibodies diluted in a solution of 1:1 Immobilon^®^ Block-Fluorescent Blocker (Cat. No. WBAVDFL01, Merk Millipore)/T-PBS buffer. The primary antibodies were: mouse anti-MITF (Cat. No. sc-515925, 1:1000, Santa Cruz Biotechnology, Santa Cruz, CA, USA), mouse anti-ER-β (Cat. No. sc-53494, 1:1000, Santa Cruz Biotechnology), rabbit anti-Zeb1 (Cat. No. sc-25388, 1:1000, Santa Cruz Biotechnology), rabbit anti-IkB (Cat. No. ab7217, 1:1000, Abcam, Cambridge, UK), mouse anti-N-Cadherin (Cat. No. M3613, 1:1000, DAKO Agilent, Milan, Italy), mouse anti-E-Cadherin (Cat. No. M3612, 1:1000, DAKO Agilent). The membrane was washed in T-PBS buffer and incubated for 1 h at room temperature with goat anti-rabbit IgG Alexa Flour 750 antibody (Cat. No. A21039; Invitrogen, Monza, Italy) or with goat anti-mouse IgG Alexa Fluor 680 antibody (Cat. No. A21057; Invitrogen), and then visualized by an Odyssey Infrared Imaging System (LI-COR^®^ Bioscience, Lincoln, NE, USA). Mouse anti-β tubulin monoclonal antibodies (Cat. No. T4026, Sigma-Aldrich) or rabbit anti-vinculin monoclonal antibodies (Cat. No. 13901, Cell signaling Technology, Danvers, MA, USA) were used to assess equal amounts of protein loaded in each lane.

### 4.4. Flow Cytometry and Cell Sorting Experiments

Cells were harvested by using Accutase (Cat. No. ECB3056D, Euroclone, Milan, Italy), and collected in flow cytometer tubes (2 × 10^5^ cells/tube). Before proceeding with primary antibody incubation, cells were permeabilized for 15 min with 0.25% Tryton X-100 PBS and then incubated for 1 h at 4 °C with an anti-ER-β antibody (Cat. No. sc-53494, Santa Cruz Biotechnology). Cells were washed in PBS and incubated for 1 h in the dark at 4 °C with secondary antibodies conjugated with FITC (Merk Millipore, Milan, Italy). Samples were washed in PBS and analyzed at BD FACSCanto (BD Biosciences, Milan, Italy). The flow cytometer was calibrated using cells incubated with secondary antibodies only. For each sample, 1 × 10^4^ events were analyzed.

For cell sorting experiments, cells were collected in microtubes (15 × 10^5^ cells/tube), fixed with cold 70% ethanol for 15 min, and permeabilized for 5 min with 0.25% Tryton X-100 PBS. Cells were incubated for 1 h at 4 °C with an anti-ERβ antibody (Cat. No. sc-53494, Santa Cruz Biotechnology), washed in PBS, and incubated for 1 h in the dark at 4 °C with a secondary antibody. Cells were then washed in PBS, re-suspended in PBS + 0.1% Trypsin + 20 × 10^−3^ M EDTA and separated using BD FACSMelody Cell Sorter (BD). High ERβ expressing (top 25%) and low ERβ-expressing (bottom 25%) subpopulations from control and acidic melanoma cells were collected according to fluorescence intensity, and RNA was extracted and analyzed as previously described.

### 4.5. siRNA Transfection

For transfections, control siRNA (siCTRL; Invitrogen, Thermo Fisher Scientific, Milan, Italy), siRNA for ESR2 (ERβ coding gene, Cat. No.14599, Invitrogen, Thermo Fisher Scientific), or siRNA for RelA (NF-κB coding gene, Cat. No. AM16708, Invitrogen, Thermo Fisher Scientific) were diluted in Optimem medium (Cat. No. 31985062, Invitrogen, Thermo Fisher Scientific). Transfections were performed using Lipofectamine 3000 reagent (Cat. No. L3000001; Invitrogen, Thermo Fisher Scientific), following the manufacturer’s instructions. In some experiments, cells were treated first with siRELA and after 24 h with siESR2.

### 4.6. Invasion Assay

Invasiveness of melanoma cells was determined in vitro in a Matrigel Basement Membrane Matrix (Cat. No. 354234; Corning, AZ, USA) on Millicell cell culture Insert (24-well PCF 8.0 μm, Millipore, Billerica, MA, USA). There were 8 × 10^4^ cells seeded in the upper compartment in 200 µL of the growth medium and incubated for 6 h at 37 °C in 10% CO_2_. After incubation, cells on the upper side of the filters were wiped off mechanically using cotton swabs and the membranes were fixed overnight in ice-cold methanol. Cells on the lower side of the membranes were stained with Diff Quick solutions and counted.

### 4.7. Statistical Analysis

Densitometric data are expressed as means ± standard errors of the mean (SEM) depicted by vertical bars from at least three independent experiments. Statistical analysis of the data was performed by Student’s *t*-test or two-way ANOVA (when more than two samples were compared). Values of *p* ≤ 0.05 were considered statistically significant.

## 5. Conclusions

Our study shows a different behavior of ERβ expression between male and female melanoma cells grown in an acidic medium and demonstrates ERβ governing EMT phenotype through an inverse relationship with NF-κB. Therefore, our results can help to understand the different sex outcomes of melanoma patients and, importantly, open new perspectives for personalized therapeutic approaches.

## Figures and Tables

**Table 1:** Primer sequences for qPCR.

Gene	Primer Forward	Primer Reverse	Amplicon Length
β-actin	CCAACCGCGAGAAGATGA	CCAGAGGCGTACAGGGATAG	97 bp
ERβ	TGATGTCCTTGACCAAGCTG	CACTTGGTCGTACAGGCTCGA	101 bp
N-Cadherin	CACTGCTCAGGACCCAGAT	TAAGCCGAGTGATGGTCC	419 bp
PTEN	TGAGTTCCCTCAGCCGTTACCT	GAGGTTTCCTCTGGTCCTGGTA	138 bp
Snail	CCCAGTGCCTCGACCACTAT	CCAGATGAGCATTGGCAG	201 bp
Twist	CGGGAGTCCGCAGTCTTA	TGAATCTTGCTCAGCTTGTC	150 bp
Vimentin	TGTCCAAATCGATGTGGATGTTTC	TTGTACCATTCTTCTGCCTCCTG	117 bp
Zeb1	GGCATACACCTACTCAACTACGG	TGGGCGGTGTAGAATCAGAGTC	155 bp
18s	CGCCGCTAGAGGTGAAATTCT	CGAACCTCCGA CTTTCGTTCT	101 bp

## Data Availability

The data generated in the present study are included in the figures and/or tables of this article.

## Supplementary Materials

The following supporting information can be downloaded at: https://www.mdpi.com/article/10.3390/ijms232315374/s1.
