# Supplementary material for: Extracellular Acidosis Differentially Regulates Estrogen Receptor β-Dependent EMT Reprogramming in Female and Male Melanoma Cells
Source: Int J Mol Sci. 2022 Dec 6;23(23):15374. doi: 10.3390/ijms232315374 (PMC9736857; doi:10.3390/ijms232315374)
Supplement: Supplementary file 1 [file ijms-23-15374-s001.zip › ijms-1971167-supplementary.pdf]

Supplementary materials

Extracellular acidosis differentially regulates estrogen receptor β-dependent EMT reprogramming in female and male melanoma cells

Silvia Peppicelli, Jessica Ruzzolini, Matteo Lulli, Alessio Biagioni, Francesca Bianchini, Adele Caldarella, Chiara Nediani, Elena Andreucci, Lido Calorini

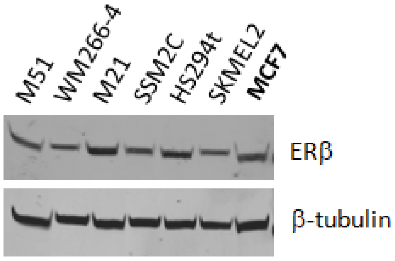

Figure S1: ERβ protein expression in melanoma cell lines; MCF7 mammary cancer cells were used as a positive control for ERβ expression.

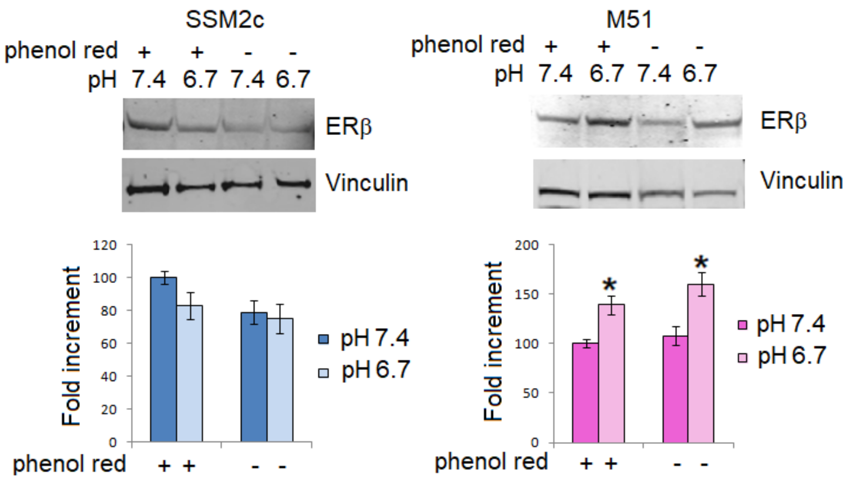

Figure S2: Western blotting analysis of ERβ protein expression in male SSM2c and female M51 melanoma cells grown for 24 hours in standard (pH 7.4) or acidic medium (pH 6.7) in DMEM with or without phenol red. Each band of the western blot was quantified by densitometric analysis and the corresponding histogram was constructed relative to vinculin. Representative Western blot panels on the top.*$p < 0.05$ compared with cells grown at standard pH**.**

Despite some small differences in ERβ expression between cells grown in DMEM with or without phenol red, melanoma cells responded to the acid treatment in a comparable way either in the presence or absence of phenol red: low pH induced ERβ expression in female M51 cells, while it did not modify ERβ expression in male SSM2c.

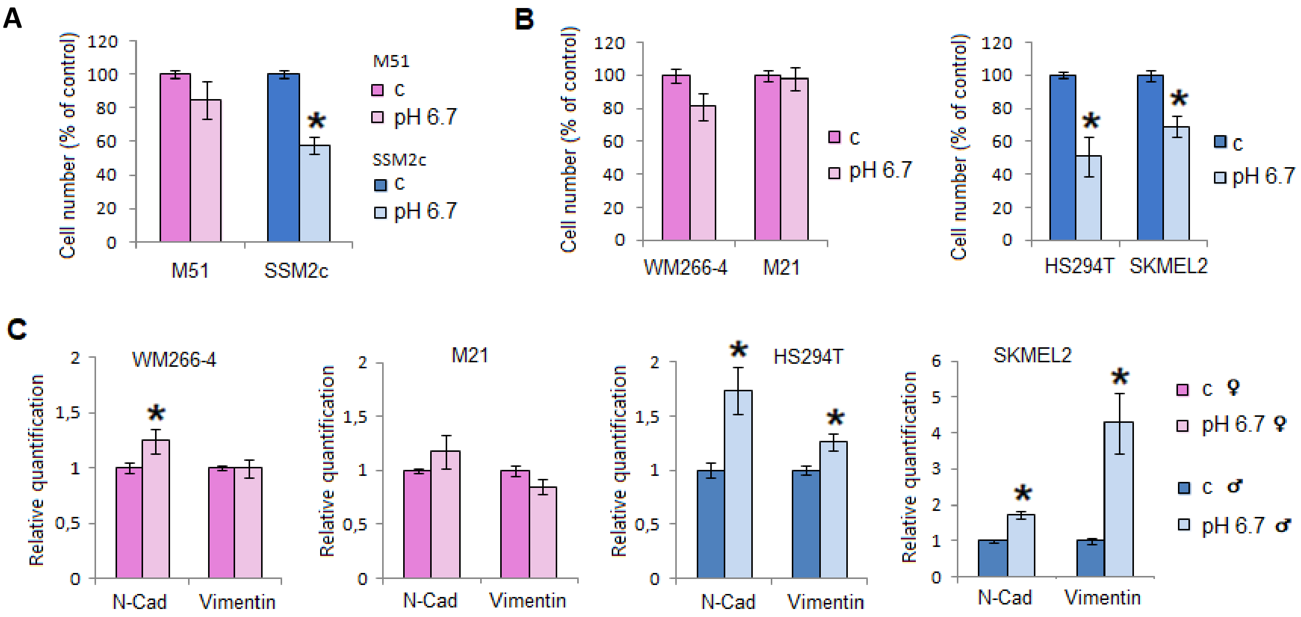

Figure S3: cell number of M51 and SSM2c (A) and WM266-4, M21, HS294t, SKMEL2 (B) melanoma cells grown for 24 hours in acidic medium. C) N-cadherin and vimentin mRNA levels in female and male melanoma cells grown for 24 hours in an acidic medium (pH 6.7). *p < 0.05 compared with control cells**.**

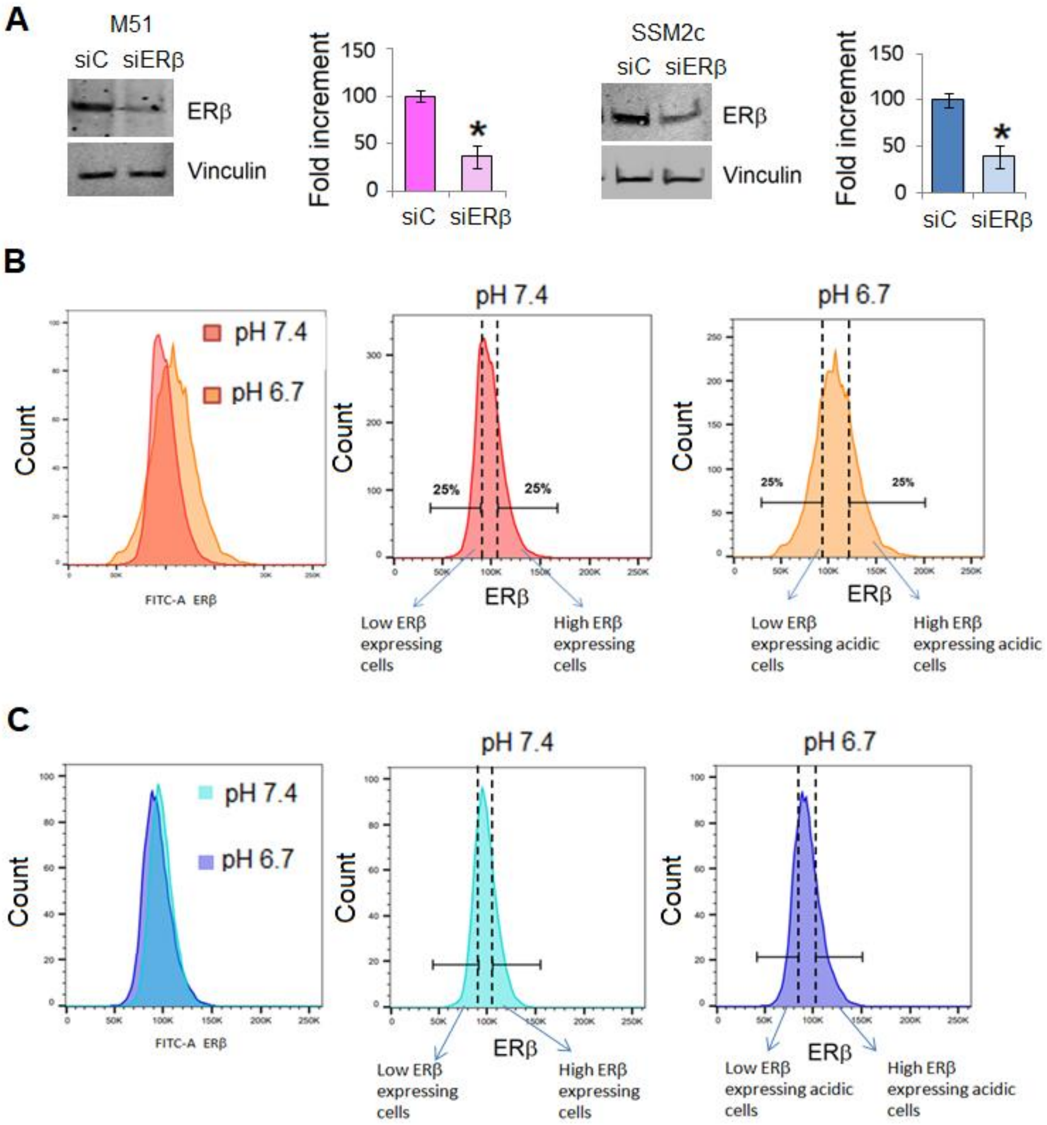

Figure S4: A) Western blotting analysis of ERβ protein expression in female M51 and male SSM2c melanoma cells silenced for ERβ. Each band of the western blot was quantified by densitometric analysis and the corresponding histogram was constructed relative to vinculin. Representative Western blot panels on the left. *$p < 0.05$ compared with control cells. Isolation of low and high ERβ expressing M51 (B) or SSM2c (C) subpopulations by flow cytometry cell sorting.

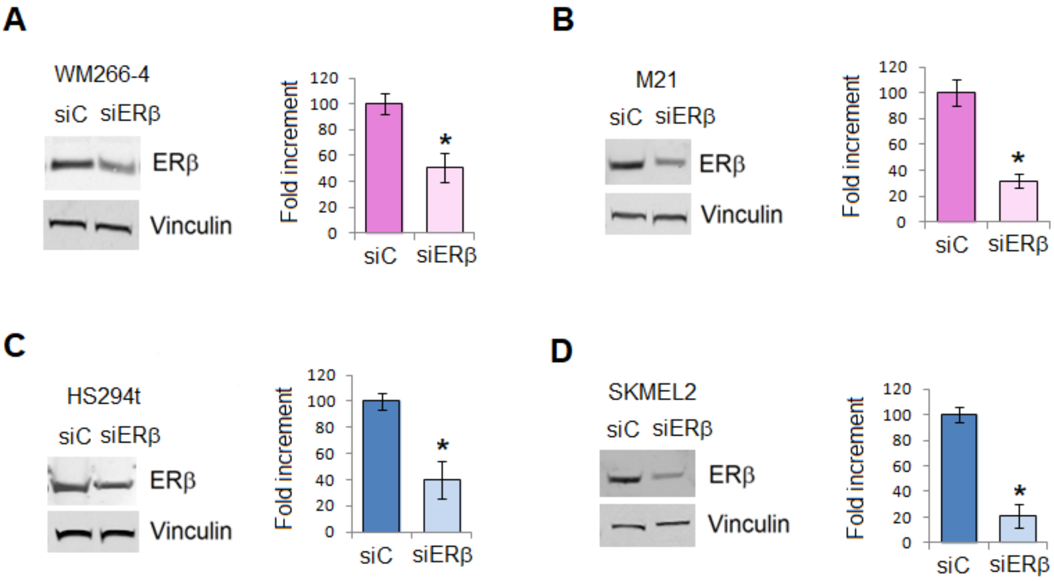

Figure S5: Western blotting analysis of ERβ protein expression in female WM266-4 (A) and M21 (B) and in male HS294t (C) and SKMEL2 (D) melanoma cells silenced for ERβ. Each band of the western blots was quantified by densitometric analysis and the corresponding histogram was constructed relative to vinculin. Representative Western blot panels on the left. *p < 0.05 compared with non-silenced cells.

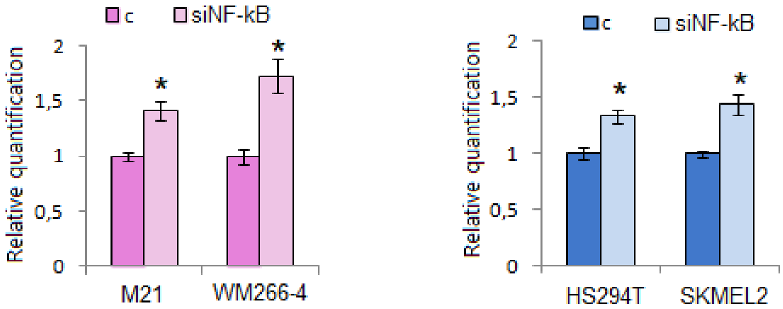

Figure S6: ERβ mRNA level of M21, WM266-4, HS294t and SKMEL2 NF-kB silenced. *p < 0.05 compared with non-silenced cells.

**Table S1.**	**Cell line**	**Metastatic site**	
	HS294t	Lymph node	From 56-year-old Caucasian male
	M21	Not specified	From female, unspecified age
	SKMel-2	Thigh; skin	From 60-year-old Caucasian male
	WM266-4	Right thigh; skin	From 55-year-old Caucasian female
**Patient-derived melanoma cells**		**Metastatic site**	
	M51	In transit metastasis, skin	From 35-year-old female
	SSM2c	In transit metastasis, skin	From 52-year-old male

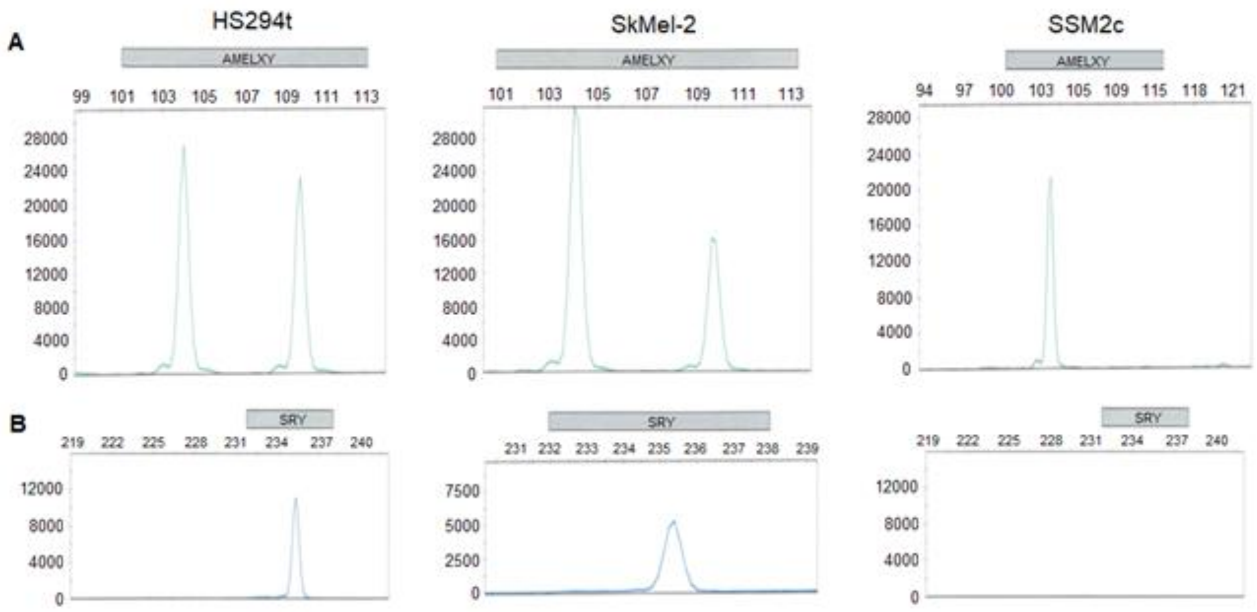

Figure S7: Electrophoretogram of the QF-PCR analysis showing the amplification products for **A)** Amelogenin (AMEL XY) and **B)** the sex-determining region of the Y chromosome (SRY**)** from the DNA of cells derived from male patients. Genomic DNA extraction was performed on the cell pellet obtained from $2x10^5$ cells, using the EZ1®DNA tissue kit (Cat. No. 953034, Qiagen, Milan, Italy) and the EZ1 instrument (Qiagen), according to the kit manufacturer's instruction. The presence of sex chromosomes was evaluated by analyzing the fluorescent QF-PCR products, obtained using the Devyser Resolution XY kit (Cat. No. 8-A012.2-XY, Devyser, HQ, Hägersten, Sweden), with the 96-capillary 3730xl DNA Analyzer (Thermo Fisher Scientific). We verified that HS294t and SKMel-2 cells conserved Y chromosome, while SSM2c cells did not show it.
